# Contextual reinstatement affects semantic organization

**DOI:** 10.3389/fpsyg.2023.1199039

**Published:** 2023-09-26

**Authors:** Dana Vaknin, Zohar Raz-Groman, Alon Scheuer, Talya Sadeh

**Affiliations:** ^1^The Department of Cognitive and Brain Sciences, Ben-Gurion University of the Negev, Beer Sheva, Israel; ^2^The School of Brain Sciences and Cognition, Ben-Gurion University of the Negev, Beer Sheva, Israel; ^3^Department of Psychology, Ben-Gurion University of the Negev, Beer Sheva, Israel

**Keywords:** episodic memory, context dependency effect, semantic organization, semantic clustering, free recall

## Abstract

The Context Dependency Effect is the well-established finding in which memory performance is enhanced under conditions in which the encoding and retrieval contexts overlap (i.e., Same-Context) and diminished when the overlap between encoding and retrieval contexts is low (i.e., Different-Context). Despite much research on context-dependent memory, most prior work examined only mean performance levels. The current experiment examined the influence of context change, manipulated by using three different pieces of background music, on semantic organization during free recall. Recall driven by semantic organization captures an important, ecologically valid aspect of memory retrieval: because narratives of real-life events are typically comprised of semantically related concepts (e.g., “sea,” “bathing suit,” and “sand” when recalling a trip to the beach), their recall is likely driven by semantic organization. Participants in the current study were tested in the same or different context as the material was learned. The results showed that although the mean number of correctly recalled items was numerically greater in the Same-Context condition compared to the Different-Context condition, the Context Dependency Effect was not significant. In contrast, however, semantic clustering—an established measure of semantic organization—was greater in the Different-Context condition compared to the Same-Context condition. Together, these results suggest that when contextual cues at recall are relatively meager, participants instead use semantic information as cues to guide memory retrieval. In line with previous findings, temporal organization, patterns of errors, and serial position analyses showed no differences between the two context conditions. The present experiment provides novel evidence on how external context change affects recall organization.

## 1. Introduction

It is well-established that episodic memory performance depends, to a large extent, on contextual reinstatement: reestablishment of the encoding context during retrieval (Tulving, 1972). Because context is associated with item/content information at encoding, it is useful in terms of cuing memory at retrieval. Therefore, memory performance is enhanced under conditions in which the encoding and retrieval contexts overlap (i.e., Same-Context), and is reduced when the overlap between encoding and retrieval contexts is low (i.e., Different-Context). This phenomenon is referred to as the Context Dependency Effect (Tulving and Thomson, 1973; Godden and Baddeley, 1975; Smith and Vela, 2001; Yonelinas, 2002). One of the most famous demonstrations of the Context Dependency Effect is the study by Godden and Baddeley (1975). In that study, the context was manipulated as the external environment in which encoding and retrieval occurred (on land or underwater). Memory performance was better when both encoding and retrieval stages occurred in the same context (either both on the ground or both underwater) than when encoding and retrieval occurred in different contexts (the former on land and the latter underwater, or vice versa). Thus, memory performance depended on whether the external context was reinstated at retrieval.

Similarly to Godden and Baddeley’s (1975) study, the majority of studies have manipulated context as a change in the features of the environment in which the experiment was conducted. Namely, under the Different-Context condition, in which encoding and retrieval occur in different contexts, the effort is made to use distinct environments. For instance, the context was manipulated as a change in orientation and illumination (Carr, 1917), use of different background music (Smith, 1985; Balch et al., 1992), change of background color (Weiss and Margolius, 1954; Rutherford, 2004), change of odor in the room (Cann and Ross, 1989; Pointer and Bond, 1998), and change in the size of the room, the furniture in it and whether or not there were windows and pictures on the walls (Smith et al., 1978). Other studies manipulated internal context—induction of a change in participants’ state of mind from the experimental context in the Different-Context condition (e.g., participants are instructed to imagine their parents’ home between study and test; Sahakyan and Kelley, 2002; Unsworth et al., 2012).

A common paradigm used in the studies examining the Context Dependency Effect is the free recall paradigm. In free recall, participants study lists of items, typically words, and recall the studied items in any order. Crucially, measures of memory performance during free recall may provide important insights into how memory is organized and searched (Bousfield and Cohen, 1953; Murdock, 1962; Puff, 1974; Kahana, 1996; Howard and Kahana, 2002b; Polyn et al., 2009). These measures include the order in which participants recall items from the studied list, as well as the likelihood of recalling items conditional on their serial position at study. Still, despite much research on context-dependent memory using free recall, most prior work examined only mean performance levels. There has been little research on how context manipulations affect measures of the dynamics of recall—relating to the temporal order of items at encoding and retrieval. Examination of recall dynamics may be of importance in understanding the Context Dependency Effect by revealing if and how context change affects memory search and organization.

The notion that examination of recall dynamics can lead to a better understanding of the Context Dependency Effect has been demonstrated by Unsworth et al. (2012). In that study, the external context was manipulated via room change, and participants were tested in the same or different context as the material was learned. In an additional experiment, internal context was manipulated by inducing a change in participants’ state of mind from the experimental context before the recall phase of the Different-Context condition. In addition to correct recall, measures pertaining to the temporal order of items at encoding and/or retrieval were examined: in particular, the serial position curve and the Temporal Contiguity, or Temporal Clustering Effect (TCE). The serial position curve measures the probability of recalling items as a function of their serial position at study. Serial position curves typically demonstrate primacy and recency effects—that is, enhanced tendency to recall items from the first and last positions on the study list. The existence and magnitude of the primacy and recency effects may be informative of the use of temporal context in driving recall (Kahana, 1996; Howard and Kahana, 1999), but see (Rundus and Atkinson, 1970; Davelaar et al., 2005) for different accounts of these effects.

The TCE refers to the finding whereby items from contiguous serial positions at study tend to be successively recalled during test (as compared to items which were remote from one another at study; Kahana, 1996; Howard and Kahana, 1999; Howard and Kahana, 2002a). According to prominent models of free recall (e.g., Temporal Context Model, TCM; and Context Maintenance and Retrieval, CMR), this effect is explained in terms of temporal context. Temporal context pertains to the thoughts and associations evoked spontaneously and by the study items. The temporal context of contiguous items is more similar to that of non-contiguous items and, therefore, recall of a certain item at test is more likely to trigger recall of an adjacent item with a similar temporal context than recall of a remote item (Howard and Kahana, 2002a). Alternate, non-contextual mechanisms for the TCE also exist. However, these do not account for all the empirical manifestations of the TCE, as contextual mechanisms do. For instance, associative chaining models ascribe the TCE to local associations between adjacent stimuli (e.g., Solway et al., 2012). These models, however, could not account for the fact that the TCE exhibits marked asymmetry (stronger tendency to recall item N + 1 following item N than followed by it), as well as findings of the TCE over both small and large timescales and across different lists (Howard and Kahana, 1999; Howard et al., 2008; Sadeh et al., 2018; Healey et al., 2019). Other models ascribe TCE to control processes (e.g., implementing encoding strategies like linking contiguous items to tell a story; Delaney and Knowles, 2005). These models, however, could not account for the finding of the TCE under incidental encoding conditions (Healey, 2018; Healey et al., 2019; Mundorf et al., 2021).

Unsworth et al. (2012) found a Context Dependency Effect for both context manipulations (external and internal), with higher recall accuracies for the Same-Context condition compared to the Different-Context condition. Interestingly, however, no differences were found between the Same-Context and Different-Context conditions in serial position curves and the TCE. These results were interpreted in terms of the search of associative memory (SAM) model (Raaijmakers and Shiffrin, 1980). According to SAM, a subset of representations in memory is activated during recall by a contextual retrieval cue. This subset of representations is related to the contextual cue and referred to as the search set. During recall, item representations are sampled from the search set based on a relative strength rule. A particular item’s probability of being sampled is determined by the degree of item-cue association (i.e., the degree of overlap between the item and contextual features). Items with the greatest relative strength, as compared with other items on the search set, will have the highest probability of being sampled. Once an item is sampled, certain features of an item become activated, and if enough of these features are activated, the item will be recovered into consciousness. Thus, recovery of an item is determined by its absolute strength. Items whose strength exceeds some critical threshold will be recovered and can be recalled, whereas weak items that do not exceed the threshold will not be recovered. When an item is recovered, participants can determine if the item was in the studied list and if so, the item will be recalled. The item which was recalled, can now be used in subsequent sampling as a retrieval cue, narrowing the search set to the information associated with this item. Accordingly, subsequent sampling is now determined by the association between items on the list. Items with strong associations with the just-recalled item have the greatest probability of being sampled.

Because the context manipulation only affected recall accuracy, but not recall dynamics, Unsworth et al. (2012) concluded that changes in context affect the recoverability of items but does not affect sampling. Changes in context lead to a reduction in the associative strengths of items. The reduction in associative strengths is due to fewer overlapping contextual features between encoded features and features present at test, making it less likely that sampled items in the Different-Context condition will actually be recovered (Unsworth et al., 2012). Based on the finding that the TCE did not differ between conditions, the authors further concluded that the context manipulation does not affect contextual bindings between items. Hence, contextual change does not affect the cuing of a certain item by prior recalled items. Importantly, however, Unsworth et al. only measured one form of cuing of items by the previously-recalled item—the TCE, which pertains to episodic, temporal context bindings between items. An abundance of studies have shown that free recall is not only driven by reinstatement of the environmental encoding context, and/or by the episodic contextual bindings between items via their overlapping temporal contexts. Rather, semantic organization also plays an important, if not crucial role, in the dynamics and probabilities of recall (e.g., Howard & Kahana, 2002b; Sirotin et al., 2005; Polyn et al., 2009). Semantic organization is typically tapped using the Semantic Clustering Effect (Polyn et al., 2009). This effect refers to the well-established finding whereby participants tend to successively recall two items that are related semantically (e.g., “KEYS” followed by “HOUSE”).

Semantic relatedness reflects previously-acquired knowledge regarding the items themselves. The importance of semantic organization in driving recall can be demonstrated by a real-life example. Consider a person telling her friend about her vacation in Greece. When recalling her vacation, the person will likely rely on prior semantic knowledge and schemas in driving recall. This, in turn, would lead to clusters of recall of semantically related concepts. For instance, she is likely to cluster together memories regarding the beaches she visited, which would include semantically related concepts such as beach, sea, sun, bathing suit and boat. To the best of our knowledge, semantic organization has not been examined in any of the previous context-effect studies. Examination of the effects of context change on semantic organization could provide invaluable insights onto the mechanisms underlying the Context Dependency Effect. Specifically, we asked whether examination of semantic organization may prompt the refinement of the theoretical proposal made by Unsworth et al. (2012), that “changes in context [do not change…] how participants use prior recalled items as cues in the next retrieval attempt.” If this theoretical proposal extends to semantic organization, no differences should be found between semantic clustering effects when the contexts at study and test are similar vs. when they are different.

On the other hand, effects of context change on semantic organization might emerge if—to rephrase the notion made above—changes in context *do* change the way the semantic representation of a just-recalled item cues the next retrieval attempt. Of particular relevance in this context is an extension of the SAM (termed eSAM) to include the effects of pre-experimental semantic knowledge on free recall (Sirotin et al., 2005). According to eSAM, each pair of items in a list are associated with one another via their (strong or weak) pre-experimental semantic relations. These pre-existing semantic associations play an important role during retrieval. Each retrieval attempt can be cued by semantic information of the previously-recalled item. Such semantic associations between items can account for semantic clustering effects. In contrast, temporal clustering, according to eSAM, is accounted for in terms of direct item-to-item associations between adjacent list items that were activated simultaneously in a short-term memory buffer. Hence, eSAM may yield different predictions with regard to experimental manipulations, such as context change, on semantic vs. temporal clustering.

An additional account of semantic clustering effects is provided by the CMR, which is an extension of the TCM to include semantic associations. CMR might also yield different predictions with regard to the effects of context change on temporal versus semantic clustering. However, CMR ascribes both temporal and semantic clustering to different mechanisms than those posited by eSAM. According to CMR, temporal clustering is driven by episodic associations formed during study. Semantic clustering, on the other hand, is a result of “longstanding context-to-item associations” (Polyn et al., 2009). According to CMR, recall of an item reinstates its entire set of pre-existing temporal contexts, namely, a blend of all previous temporal contexts the item has been associated with. The pre-existing temporal contexts of semantically-similar items largely overlap with one another, hence cuing retrieval of each other. Importantly, a simulation study investigating the mechanisms underlying semantic organization in recall found that semantic associations between items (as stipulated in eSAM) are more likely to account for semantic clustering effects than context-to-item associations (as stipulated in CMR; Morton and Polyn, 2016). Thus, semantic clustering should be sensitive to manipulations affecting the contribution of semantic associations between items to recall.

We predicted that semantic clustering may be affected by context change if this manipulation changes the relative contribution of different cues to memory. In the Different-Context condition, item-context associations may be less effective (compared to the Same-Context condition) because there are fewer overlapping contextual features between encoding and retrieval. Instead, semantic associations between items might play a more prominent role. Thus, if indeed context change exerts an effect on semantic organization, this might not necessarily be a detrimental one. Effects of context change on semantic organization might be similar to the effects of the passage of time. In both context change manipulations and delay over time, the context at retrieval is less similar to the context at encoding (compared to Same-Context and immediate retrieval, respectively; Sederberg et al., 2008). A study by Gamoran et al. (2020) examined the effect of delay-dependent forgetting on various measures of free recall, including semantic clustering. Semantic organization was found to be significantly higher in the long delay condition than in the short delay condition. This result was explained in terms of the relatively larger contribution of semantic cues after delay, when fewer contextual cues are available. If context change exerts similar effects on semantic organization as delay over time, a greater semantic clustering effect might be found in the Different-Context condition than in the Same-Context condition.

In sum, different patterns of results regarding the effects of context on semantic organization may emerge, each with different theoretical implications. First, in line with the theoretical proposal of Unsworth et al. (2012), context change may not affect semantic clustering, because it does not change the way in which one item cues retrieval of the next. On the other hand, semantic clustering may be affected (negatively or positively) by context change if such a manipulation does affect the cuing of an item by the previously-recalled item, as elaborated on above. The current experiment aimed to elucidate between these two possibilities by examining whether there is a difference in semantic clustering when the contexts at encoding and retrieval are the same compared to when there are different contexts at encoding and retrieval. We manipulated the degree of semantic relatedness between the words in the study lists. Importantly, this manipulation was designed to increase the ecological validity of our paradigm (compared to paradigms using random word lists), because narratives of real-life events are comprised of semantically related concepts, as was illustrated in the example above. In previous studies of the Context Dependency Effect, completely random lists of words, which were semantically unrelated, were studied and recalled. Therefore, participants in those studies could not rely on semantic organization to drive recall.

## 2. Methods

### 2.1. Participants

A total of 250 Amazon Mechanical Turk (Mturk) workers (ages 19 to 39) participated in the experiment. The study took an average of 20 min and 34 s, and respondents were paid 1.50$ for completing the study. In addition, Prolific workers (ages 19 to 39, M = 30.8, SD = 5.1) took part in the control group of this experiment. The control group took an average of 18 min and 58 s to complete the experiment. Participants read and signed informed consent of willingness to participate in the study. The informed consent specified that participants would be compensated for their participation if they completed the study and correctly answered quality check questions. Quality check questions were added to the study to filter out automatically filling “bots” and inattentive participants (see Materials). Participants were excluded if they met one of the following *a-priori* exclusion criteria: (a) Incorrect answers to one or more of three quality check questions. (b) Failure to fully complete the study. These exclusion criteria resulted in the exclusion of data from 109 Mturk participants and three Prolific participants. Thus, analyses were conducted on 141 participants in the main experiment and 58 participants (26 female) in the control experiment.

The Human Subject Research Committee at Ben-Gurion University approved the study.

### 2.2. Materials

The experiment included three lists of 32 words each. For each participant, two lists were used during the experiment as study lists, and one was used during the practice phase (see Experimental Procedure below). Words were 3–10 letters long nouns and adjectives, selected from the Penn Electrophysiology of Encoding and Retrieval Study (PEERS) word pool, which contains 1,638 words.^1^ The lists were constructed such that temporal and semantic contributions to recall can be dissociated. In each list, varying degrees of semantic relatedness occurred at adjacent and distant serial positions. Semantic relatedness was determined using the Word Association Space (WAS) model described by Steyvers et al. (2004). WAS similarity values were used to identify pairs of words that shared high similarity (cosθ>0.7), such as PILOT-AIRPLANE and ELECTRON-ATOM. For each list, 16 pairs of words with high semantic similarity were selected. Next, words within the lists were organized such that members of a pair did not appear adjacent to one another. Four different ordering schemes of the words within the list were created for each list, such that no adjacent pair of words was of high semantic similarity. Each participant was randomly assigned to one of these ordering schemes.

The main experiment was built using the OpenSesame platform and run on the web. It was distributed using MTurk. The control experiment was built using Javascript and run on the web as well. It was distributed using Prolific.

#### 2.2.1. Context manipulation

The context was manipulated using three music types: classical, jazz, and meditation. One piece was selected from each music type and played as background music to the participants during the experiment. The choice of classical and jazz music followed previous studies (Balch et al., 1992). We chose meditation music as a third type of music. The background music contained no lyrics to prevent interference with the mnemonic processing of the verbal stimuli used in the study. The musical pieces were trimmed to fit the length of the experiment.

The background music began playing at the start of each phase (encoding, retrieval) and stopped playing at the end of each phase. There was a pause between the phases in which no music was played. In the Same-Context condition, the same background music (e.g., A) was played for both the encoding and retrieval phases. The music in the retrieval phase restarted from the beginning of the recording. In the Different-Context condition, one type of background music (e.g., B) was played during the encoding phase and another (e.g., C) during the retrieval phase. The order of the conditions was counterbalanced across participants. The assignment of context (A, B, C) to condition was counterbalanced across participants. In the control group, no music was played during the experiment.

### 2.3. Experimental procedure

**Figure 1:**
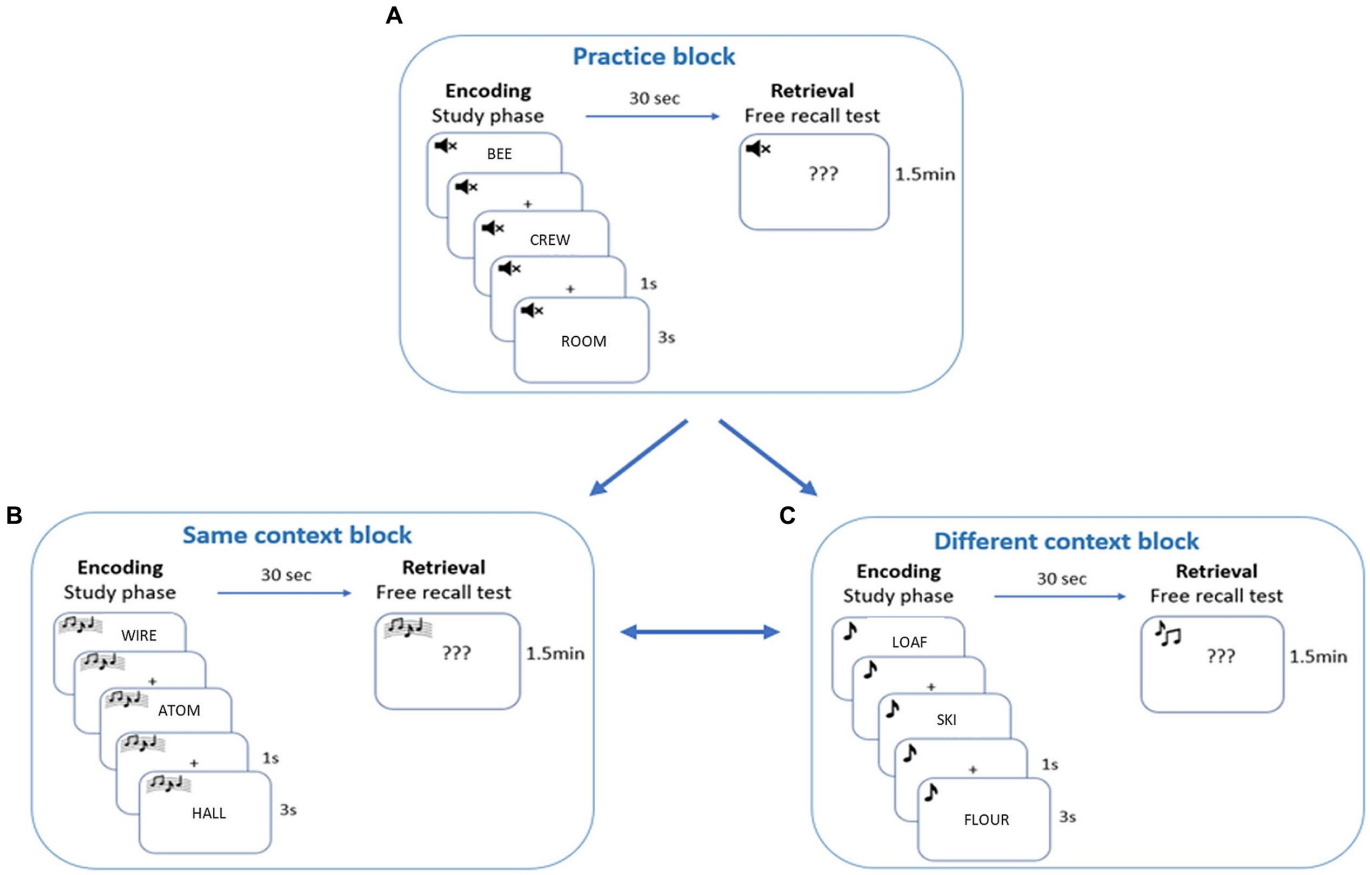
Illustration of the experimental procedure for a single participant. During encoding, 32 words were presented, one after the other and participants were required to learn the words for a subsequent free recall test. At retrieval, participants tried to recall as many words as possible. In the practice block, no background music was played during the encoding and retrieval phases. In the Same-Context block, the same background music was (e.g., **A**) played during the encoding and retrieval phases. In the Different-Context block, one background music was played during the encoding phase (e.g., **B**) and another during the retrieval phase (e.g., **C**). The order of the conditions was counterbalanced across participants. The assignment of context **(A–C)** to condition was counterbalanced across participants.

The main experiment included one within-participants independent variable with two conditions: Same-Context condition and Different-Context condition. The experiment began by giving detailed instructions to the participants and consisted of three blocks: practice block, Same-Context block, and Different-Context block. The order of Same-Context and Different-Context blocks was counterbalanced across participants. Each block consisted of two phases: (a) the encoding phase and (b) the retrieval phase. Figure 1 illustrates the experimental procedure for a single participant. To establish that the results obtained in the main experiment were not due to the general effects of music on memory performance, a control experiment with the same procedure was conducted, with the exception that no music was included at any stage.

In both experiments, participants were instructed to perform the study on a computer, and avoid using tablets or phones. At the beginning of the study, participants were asked to turn on the speakers and raise the volume.

#### 2.3.1. Encoding phase

In each encoding phase, 32 words were presented visually in the middle of the screen for 3,000 ms, followed by a fixation cross that appeared on the screen for 1,000 ms. Participants were requested to remember as many words as possible for a subsequent memory test. Background music was played throughout the encoding phase.

#### 2.3.2. Retrieval phase

Following the encoding phase, instructions on the screen requested participants to type in all the words they could recall from the most recent list, one word at a time, in any order. The total duration of this phase was 90,000 ms. Participants typed their responses and pressed Enter after each word they typed. Following each response, the screen went blank, and participants could type the next word they recalled. Background music was played during the retrieval phase.

#### 2.3.3. Practice block

This block was conducted prior to the experimental blocks (Same-Context/Different-Context). It included one practice list of 32 words and a free recall test phase, to familiarize participants with the task. During the encoding phase and the retrieval phase of this block, no background music was played. Data from this block were not analyzed.

#### 2.3.4. Quality check questions

We included three quality check questions to filter out bots and to ensure that online participants completed the experiment adequately. The first was given before the practice list. Participants were asked to listen to a short ambiguous text: “The trophy does not fit into the brown suitcase because it is too small” and answer the following question: “What is too small?.” This question aimed to make sure that (a) participants were paying attention to the instructions and (b) participants had their device’s sound turned on so they could hear the background music during the experiment, (c) “bots” that are presumably unable to interpret the ambiguity in the question and answer it correctly were filtered out. The second quality check, with the same aims as the first, was presented at the end of the second list’s retrieval phase. Participants were asked to listen to another short text: “A large ball crashed right through the table because it was made of steel,” and answer the following question: “What was made of steel?”

The third quality check was right after the second; participants were asked to answer the question: “what type of music was played during the experiment?” by typing one word that describes the music they heard. This question ensured that participants listened to the background music played throughout the experiment. The answers to these questions were manually evaluated and rejected if they did not match the music that was played. Participants were excluded from the analyses if they failed to answer at least one of the quality check questions correctly.

### 2.4. Data analysis

All data were processed with in-house Matlab scripts (The Mathworks, Natick, MA, United States) and R scripts (R Core Team, 2021). Scripts for the temporal and semantic clustering effects were based on those from the Kahana lab.^2^ Statistical analyses, including Bayesian analyses, were performed with JASP version 0.9 (JASP Team, 2020) and with R version 4.3.0. When violation of the equal variances assumption occurred, the degrees of freedom of the unpaired t-tests were adjusted using the WelchSatterthwaite method. In R, data was organized and analyzed using the tidyverse package (Wickham et al., 2019). Plots were produced using the ggpubr package (Kassambara, 2022a). Statistical tests in R were performed using the rstatix package (Kassambara, 2022b). Participants’ responses at the test phase were extracted and, using the hunspell package (Ooms, 2022), were corrected for misspelled responses.

#### 2.4.1. Temporal organization

Lag conditional-response probabilities (lag-CRPs; Kahana, 1996) were calculated for each participant in each condition (Same-Context, Different-Context). Given a sequence of recalled items, we categorized each transition during the retrieval phase according to the temporal distance between the two items in the encoding phase. Each transition is coined ‘lag’. For example, if a participant recalled the following items (numbered according to their serial position during the encoding phase): 5, 6, and 2, their respective lags are 1 and − 4. After scoring all the actual transitions according to their lags, we also scored all possible transitions. Possible transitions exclude transitions to items already recalled or lags that exceed the list’s length (e.g., if item 32 was recalled, a lag of +1 is not possible). For each lag value, the conditional-response probability was the amount of times it appeared (during recall) divided by the amount of times it was possible during recall.

The Temporal Factor score is a single value that measures participants’ tendency to recall two words at short lags successively (Sederberg et al., 2008). Lag refers to the distance between the serial positions of the two recalled words. The absolute values of the lags of all actual and possible transitions between two recalled words were calculated. All possible transitions were given a Spearman’s rank based on the absolute lag (with the lowest lag given the highest rank). Following this transformation, each transition received a Temporal Factor Score between 0 and 1 based on the following equation: R-1/ N-1, where R is the rank of the actual transition made, and N is the number of possible transitions that could have been made. The transition scores were then averaged to yield two scores per participant: one for each context condition. These scores have values between 0 and 1, with a score of 0.5 indicating that half of the time a participant made temporally contiguous transitions and half of the time s/he did not, thus indicating chance-level organization, which did not take into account the temporal organization. A Temporal Factor score of 1 indicates that all the transitions received the highest possible score regarding the temporal organization.

#### 2.4.2. Semantic organization

To explore semantic memory organization, individual Semantic Factor scores were calculated. A similar calculation was performed as that used for the Temporal Factor Scores. Each transition between two recalled words also received a Semantic Factor score between 0 and 1 based on the following equation: R-1/N-1. In this case, N refers to the number of all possible transitions that could have been made between two words in the list, and R to the rank of the actual transition in terms of semantic distance between the two words recalled. Similarly to Temporal Factor Scores, Semantic Factor scores have a value between 0 and 1, with a score of 0.5 signifying a chance level of semantic organization (Sederberg et al., 2010).

#### 2.4.3. Patterns of errors

Incorrect recalls included Extra-List Intrusions (ELIs): words not studied in any list.

## 3. Results

### 3.1. Correct recall

On the descriptive level, the results support the Context Dependency Effect. The number of correctly recalled words in the Same-Context condition (M = 12.496, SD = 5.740) was larger than in the Different-Context condition (M = 12.121, SD = 5.474). However, this difference did not reach a statistical level of significance. A mixed effects ANOVA was conducted on correct recall, with the effect of context condition (Same-Context, Different-Context) as a within-subjects factor and condition order (Same-Context first and then Different-Context or vice versa) as a between-subject factor. The ANOVA revealed no significant effect of context condition [*F* (1,139) = 1.555, *p* = 0.215, η^2^_p_ = 0.001], and no significant interaction between context condition and condition order [F (1,139) = 2.575, *p* = 0.509, η^2^_p_ = 2.920e-4].

In the control experiment, where no music was included, the mean number of correctly recalled items was between the means of the two context conditions (M = 12.345, SD = 5.932). It was not significantly different from the mean of the Different-Context condition [*t* (237.02) = 0.312, *p*_bonf_ = 1] nor from the mean of the Same-Context condition [*t* (242.32) = −0.207, *p*_bonf_ = 1].

### 3.2. Semantic organization

**Figure 2:**
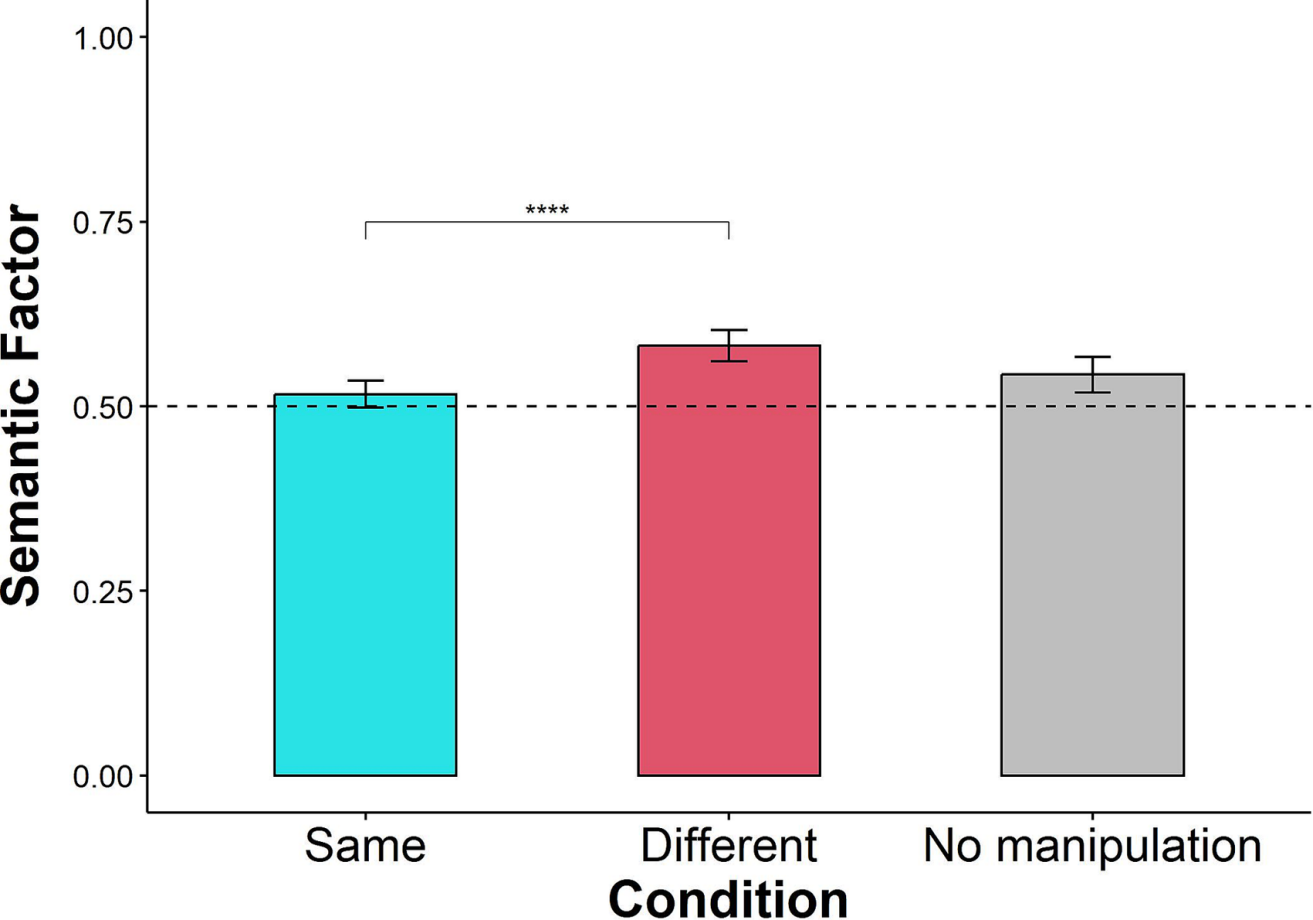
Semantic Factor scores per condition. Error bars represent 95% confidence intervals around the mean. ****p < 0.0001.

The values of the Semantic Factor scores are presented in Figure 2. The mean Semantic Factor scores for the Same-Context and the Different-Context conditions were greater than chance (0.5). For the Same-Context condition, the Semantic Factor score was 0.516 {SD = 0.106, *t* (134) = 1.802, *p* = 0.037, *Cohen’s d* = 0.155, 95% CIs [0.501, Inf]}, and for the Different-Context condition, the Semantic Factor score was 0.582 {SD = 0.125, *t* (134) = 7.65, *p* < 0.001, *Cohen’s d* = 0.658, 95% CIs [0.564, Inf]}. Thus, participants tended to recall words that were semantically related successively.

On the descriptive level, the mean Semantic Factor score in the Different-Context condition was higher than in the Same-Context condition. A mixed effects ANOVA was conducted on the Semantic Factor scores, with the effect of context condition (Same-Context, Different-Context) as a within-subjects factor and condition order (Same-Context first and then Different-Context or vice versa) as a between-subject factor. The ANOVA revealed a significant effect of context condition [*F* (1,131) = 25.960, *p* < 0.001, η^2^_p_ = 0.072], no significant effect of condition order [F (1,131) = 1.019, *p* = 0.315, η^2^_p_ = 0.004], and no significant interaction between context condition and condition order [F (1,131) = 0.351, *p* = 0.555, η^2^_p_ = 9.674e-4]. This finding demonstrates that semantic organization is sensitive to changes in context and that the order of conditions does not affect Semantic organization.

In the control experiment, the Semantic Factor was also above chance, with a mean of 0.543 {SD = 0.128, *t* (114) = 3.574, *p* < 0.001, *Cohen’s d* = 0.333, 95% CIs [0.523, Inf]}. It was also significantly lower than the mean of the Different-Context condition {*t* (239.46) = −2.45, *p*_bonf_ = 0.03, *Cohen’s d* = 0.311, 95% CIs [−0.071, −0.008]}. It was not significantly higher than the mean of the Same-Context condition {*t* (220.82) = 1.76, *p*_bonf_ = 0.16, *Cohen’s d* = 0.225, 95% CIs [−0.003, 0.056]}.

### 3.3. Correlation between correct recall and semantic organization

Having demonstrated that participants rely on semantic organization to drive their responses, we sought to determine whether reliance on semantic organization is correlated with recall performance, specifically if higher reliance on semantic organization is associated with greater recall performance.

A Pearson’s correlation coefficient was computed to assess the linear relationship between correct recall and semantic organization for each context effect condition (Same-Context and Different-Context) and in the control experiment. The Pearson’s correlations were insignificant for the Same-condition {*r* (133) = −0.073, *p* = 0.402, 95% CIs [−0.239, 0.097]}, insignificant for the Different-condition {*r* (133) = −0.009, *p* = 0.913, 95% CIs [−0.178, 0.16]}, and insignificant for the control experiment {*r* (56) = 0.060, *p* = 0.651, 95% CIs [−0.201, 0.314]}.

### 3.4. Lag conditional-response probability

**Figure 3:**
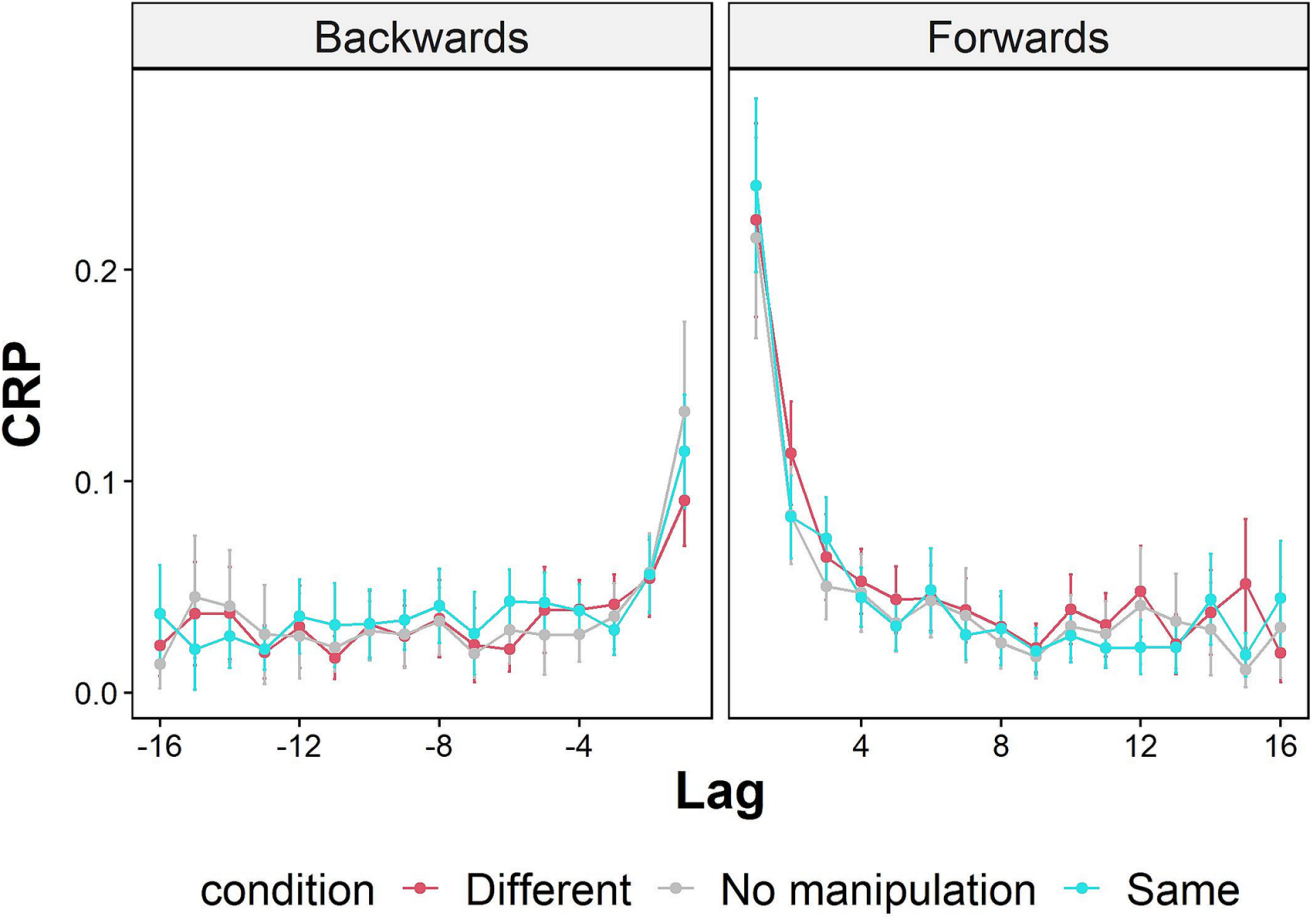
Conditional response probability (CRP) functions for forward and backward transitions as a function of temporal lag (lag) and context condition (Same-Context, Different-Context, and control), calculated across subjects. Error bars represent 95% confidence intervals around the mean.

Lag conditional-response probability curves were calculated for each context condition and are shown in Figure 3. Visual examinations of the lag-CRP figure show that for both context conditions, after recalling an item, participants tended to recall items from the first and second nearby list positions, compared with the rest of the serial list positions. To examine differences between the lag-CRP curves in the two context conditions, we followed previous research (Golomb et al., 2008) and averaged lag 1 and lag 2 transitions into one group defined as adjacent transitions lags, and compared it with the average of the larger lags (lag 3–lag 16), at both forward and backward lags, defined as remote transition lags.

A mixed-effects ANOVA was conducted on conditional-response probability scores, with context condition (Same-Context, Different-Context), lag proximity (adjacent, remote), and lag direction (forward, backward) as within-subject factors and condition order (Same-Context first and then Different-Context or vice versa) as a between-subject factor. There was a significant bias toward forward lags, confirmed by a significant main effect of lag direction [*F* (1,127) = 33.845, *p* < 0.001, η^2^_p_ = 0.035]. There was also a significant main effect of lag proximity [*F* (1,127) = 161.327, *p* < 0.001, η^2^_p_ = 0.197], expressing the bias toward adjacent, compared to remote lags. In addition, the two-way interaction between lag direction and lag proximity was significant [*F* (1,127) = 20.302, *p* < 0.001, η^2^_p_ = 0.023]. Thus, a significant asymmetry effect was exhibited, whereby adjacent lags are more likely to be in the forward direction than remote lags.

There were no differences between the context conditions regarding the lag-CRP function. Specifically, the two-way interaction between context condition and lag proximity was insignificant [F (1,127) = 2.520, *p* = 0.115, η^2^_p_ = 8.855e-4]. Likewise, the interaction between context condition and lag direction was insignificant [F (1,127) = 0.657, *p* = 0.419, η^2^_p_ = 2.905e-4]. The three-way interaction between context condition, lag direction, and lag proximity did not reach significance [*F* (1,134) = 0.028, *p* = 0.866, η^2^_p_ = 2.121e-4]. The interaction between condition order, lag direction, lag proximity, and condition order was also insignificant [F (1,127) = 0.218, *p* = 0.099, η^2^_p_ = 0.001], indicating that the order of conditions did not affect the three-way interaction.

### 3.5. Temporal factor score

The mean Temporal Factor scores for the Same-Context and Different-Context conditions were greater than chance (0.5). For the Same-Context condition, the Temporal Factor score was 0.672 {SD = 0.165, *t* (134) = 12.047, *p* < 0.001, *Cohen’s d* = 1.037, 95% CIs [0.648, Inf]}, and for the Different-Context condition, the Temporal Factor score was 0.668 {SD = 0.17, t (134) = 11.421, *p* < 0.001, *Cohen’s d* = 0.983, 95% CIs [0.643, Inf]}. Thus, participants tended to recall words from contiguous serial positions successively.

A mixed effects ANOVA was conducted on the Temporal Factor score, with the effect of context condition (Same-Context, Different-Context) as a within-subjects factor and condition order (Same-Context first and then Different-Context or vice versa) as a between-subject factor. The ANOVA revealed no significant effect of context condition [*F* (1,131) = 0.036, *p* = 0.851, η^2^_p_ = 6.617e-5], no significant effect of condition order [F (1,131) = 0.910, *p* = 0.342, η^2^_p_ = 0.005], and no significant interaction between context condition and condition order [F (1,131) = 0.036, *p* = 0.851, η^2^_p_ = 6.617e-5].

In the control experiment, the Temporal Factor was also above chance with a mean of 0.648 {SD = 0.193, *t*(114) = 8.224, *p* < 0.001, *Cohen’s d* = 0.767, 95% CIs [0.618, Inf]}. It was not significantly lower than the mean of the Different-Context condition {*t* (229.83) = −0.856, *p*_bonf_ = 0.786, *Cohen’s d* = 0.109, 95% CIs [−0.066, 0.026]} nor than the mean of the Same-Context condition {*t* (226.31) = −1.04, *p*_bonf_ = 0.598, *Cohen’s d* = −0.133, 95% CIs [−0.069, 0.021]}.

### 3.6. Serial position curves

**Figure 4:**
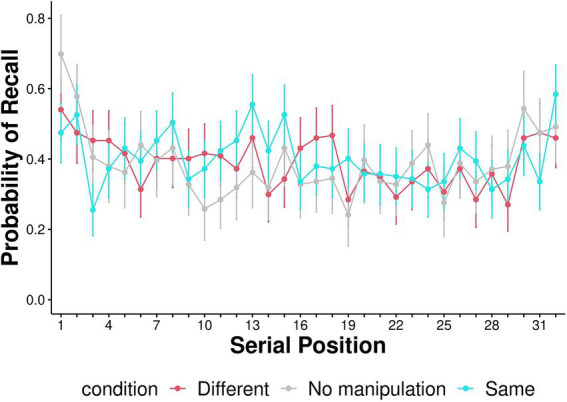
The serial position curve—the proportion of words recalled from each serial position (out of all serial positions) in each condition. Error bars represent 95% confidence intervals around the mean.

**Figure 5:**
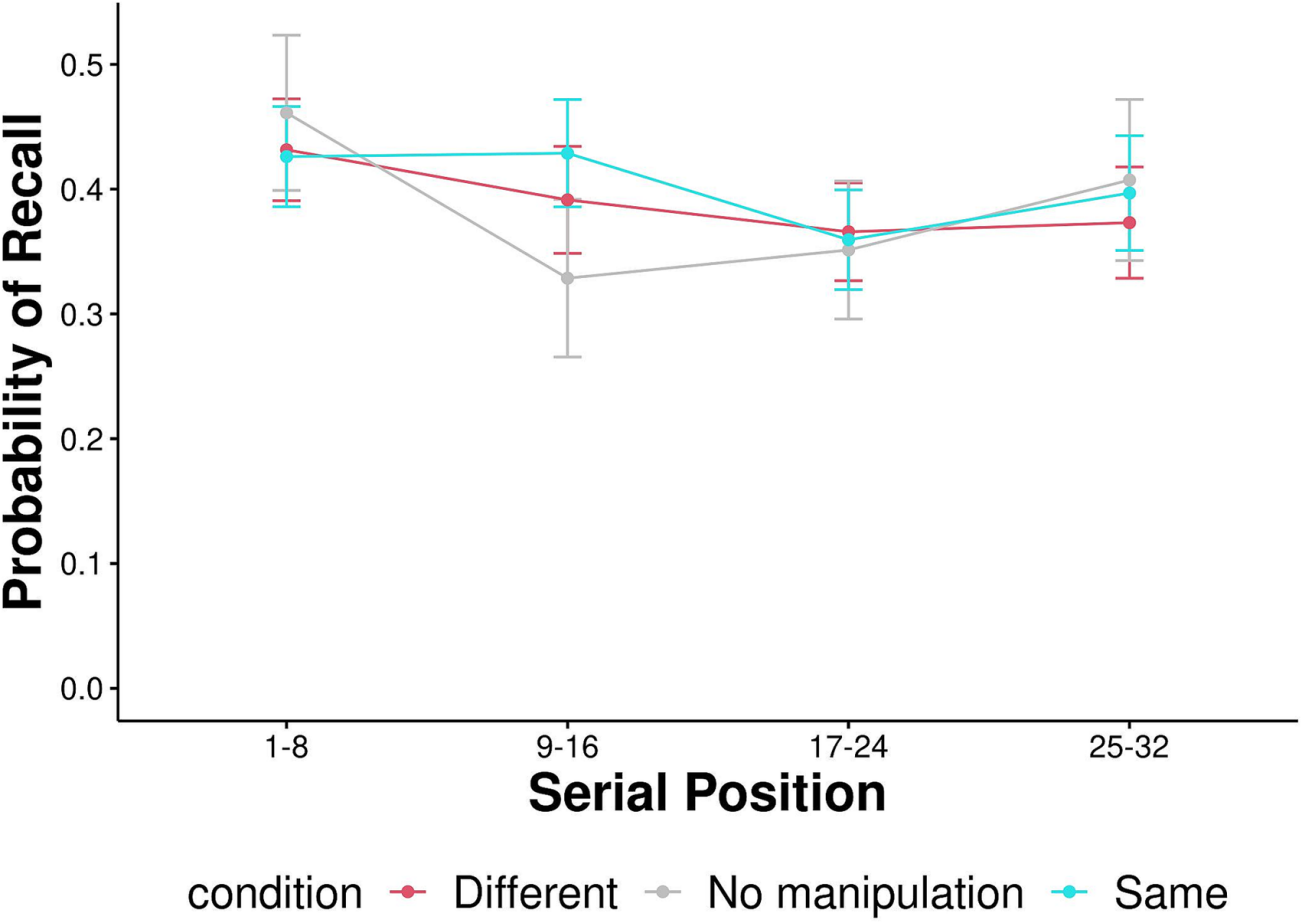
The serial position curve is split into four serial position bins for each context condition. The probability of recall in each bin in each context condition. Error bars represent 95% confidence intervals around the mean.

**Figure 6:**
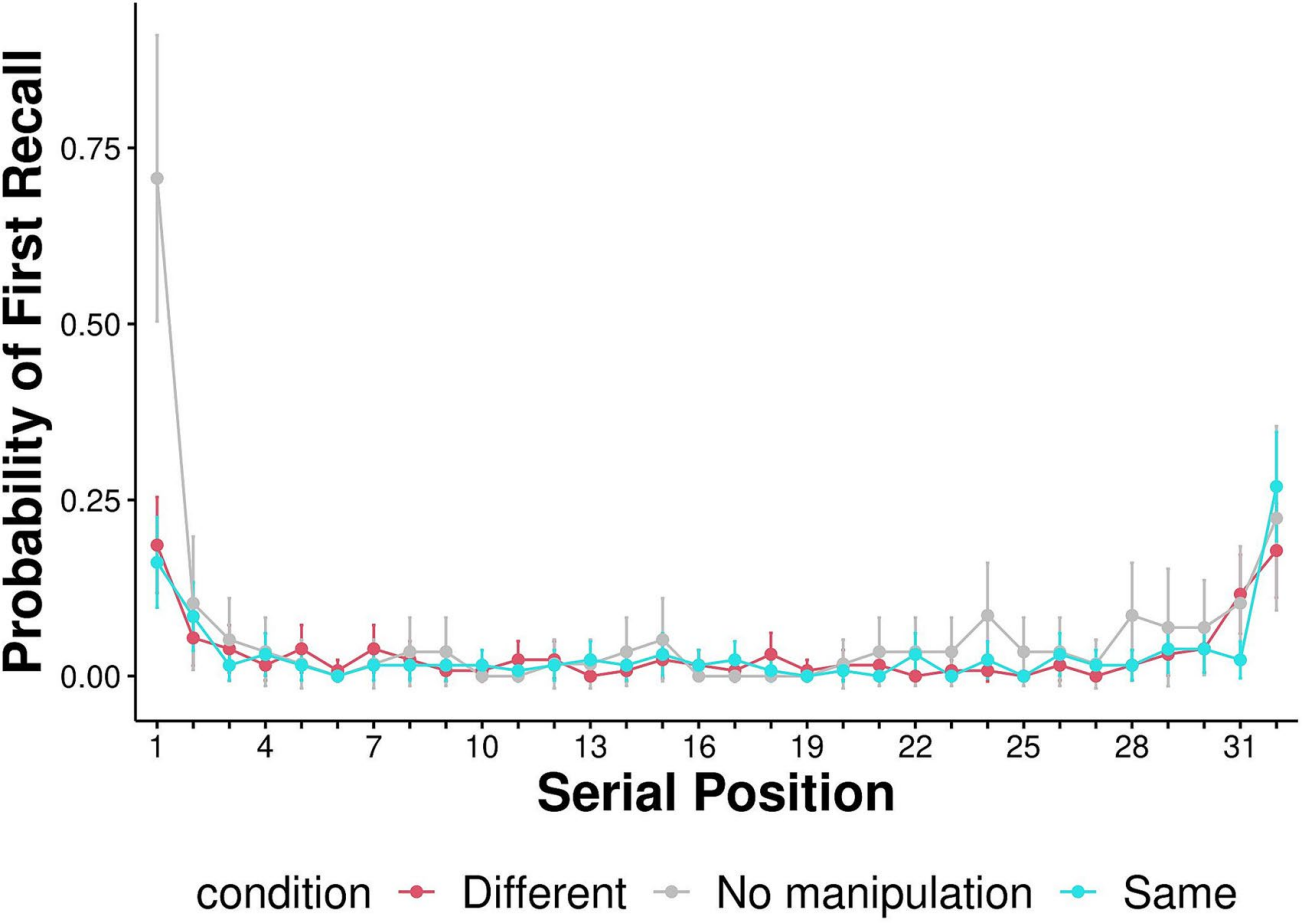
Probability of first recall—the probability of the first item recalled being from each of the serial positions. Error bars represent 95% confidence intervals around the mean.

Serial position curves are presented in Figure 4. We followed previous research to examine the differences in serial position curves between the context effect conditions (Same-Context and Different-Context; Brooks, 1999). We split the 32 serial positions into four equal bins, each containing eight positions (1–8, 9–16, 17–24, 25–32; Figure 5). A mixed effects ANOVA was conducted with context condition (Same-Context, Different-Context) and serial position (1–8, 9–16, 17–24, 25–32) as within-subject factors and condition order (Same-Context first and then Different-Context or vice versa) as a between-subjects factor. A Greenhouse–Geisser adjustment was used to correct violations of sphericity in the ANOVA. The ANOVA revealed a significant effect for Bin [*F* (2.676, 399.049) = 5.131, *p* = 0.003, η^2^_p_ = 0.020]. No significant main effect was found for the context condition [*F* (1,139) = 0.506, *p* = 0.478, η^2^_p_ = 2.384e-4]. The interaction between the context condition and Bin was insignificant [*F* (2.871, 399.049) = 1.634, *p* = 0.183, η^2^_p_ = 0.004]. The three-way interaction between context condition, Bin, and condition order was significant [F (2.871, 399.049) = 2.986, *p* = 0.033, η^2^_p_ = 0.007]. However, Tukey *post-hoc* tests revealed no significant differences between any of the conditions. Thus, participants in two context conditions demonstrated broadly similar serial position functions. Furthermore, condition order did not affect the serial position function. To further elucidate recall dynamics and in line with Unsworth et al. (2012), probability of first recall is represented in Figure 6.

Interestingly, though there are no differences in serial position curves between any of the conditions, the probability of first recall is substantially higher in the no manipulation condition than in both the Same- and Different-context conditions. While additional research is needed to determine whether this result is robust, it raises the possibility that music affects recall initiation. This might be because the music (whether the same or different from that during encoding) distracts participants from reinstating the beginning-of-list context at the onset of recall. Still, this context is reinstated to the same degree in all conditions at some point during the recall session, as evident by the findings of no differences in serial position curves between the conditions. Another potential reason for differences in probabilities of first recall is possible differences in the populations from which we sampled our participants. Participants in the Same- and Different-Context conditions were recruited from the Amazon Mturk pool and the No-Manipulation participants were recruited from Prolific. Importantly, differences between the samples, if such exist, were not reflected in any other mnemonic measure, including overall recall performance, temporal organization and serial position curves.

### 3.7. Patterns of errors

In the Different-Context condition, participants had numerically more ELIs (M = 0.794, SD = 1.32) than in the Same-Context condition (M = 0.681, SD = 1.59). A mixed effects ANOVA was conducted on the ELIs, with the effect of context condition (Same-Context, Different-Context) as a within-subjects factor and condition order (Same-Context first and then Different-Context or vice versa) as a between-subject factor. The ANOVA revealed no significant effect of context condition [*F* (1,139) = 1.008, *p* = 0.317, η^2^_p_ = 0.001], no significant effect of condition order [*F* (1,131) = 0.150, *p* = 0.699, η^2^_p_ = 8.944e-4], and no significant interaction between context condition and condition order [F (1,131) = 2.613, *p* = 0.108, η^2^_p_ = 0.003].

In the control experiment, the mean number of ELIs was between the means of the two context conditions (M = 0.698, SD = 1.19), and not significantly different than either of them {control-different: *t* (237.02) = 0.312, *p*_bonf_ = 1, *Cohen’s d* = 0.039, 95% CIs [−1.191, 1.639]; control-same: *t* (242.32) = −0.207, *p*_bonf_ = 1, *Cohen’s d* = −0.026, 95% CIs [−1.595, 1.292]}.

## 4. Discussion

The current study aimed to examine the effects of context change on the dynamics of free recall. Specifically, the main goal was to determine whether there is a difference in the reliance on a semantic organization when the contexts at encoding and retrieval are the same, compared to when the contexts at encoding and retrieval are different from one another. To this end, participants were tested in the same or different context as the material was learned, using different background music in a within-subject study design. The degree of semantic relatedness between the words in the learning lists was manipulated. Our main question concerned the effects of context change on semantic organization in recall. Semantic Factor scores were above chance in both context conditions, indicating that participants used semantic clustering during recall in the Same-Context and Different-Context conditions. However, the Semantic Factor score was significantly higher in the Different-Context condition compared to the Same-Context condition, indicating that participants relied more on semantic organization when the context presented at retrieval differed from the context presented at encoding.

According to the notion that memory performance depends on the reinstatement of the encoding context during retrieval, participants in the Same-Context condition should recall significantly more words than those in the Different-Context condition. Indeed, in the present experiment, participants recalled more words in the Same-Context condition. However, the difference did not reach statistical significance. This may be due to the small effect size of Context-Dependency (*d = 0*.28; Smith and Vela, 2001). Indeed, although the Context Dependency Effect is seminal in memory research, the beneficial effect of context reinstatement has not been consistently supported in the memory literature, and many studies failed to elicit the effect (Fernandez and Glenberg, 1985; Wälti et al., 2019). This is particularly true for studies which used recognition paradigms, but a failure to find Context Dependency Effects, or weak effects, have also been reported in free recall (Izawa, 2014). According to the mental reinstatement hypothesis, this might be due to the fact that participants in free recall experiments are typically instructed to mentally reinstate the study context, and that participants are able to do this for both the Same- and the Different-context conditions equally well (Bjork and Richardson-Klavehn, 1989). A different interpretation of the failure to find a Context Dependency Effect is the outshining hypothesis. According to this hypothesis, the effectiveness of the environmental, external context cues to prompt memory depends on the other available cues, which in certain cases might outshine the external context cues (Bjork and Richardson-Klavehn, 1989). This hypothesis might provide an interpretation to the weak Context Dependency Effect in the current study: the semantic cues outshone the environmental cues.

The current results imply that not only did semantic cues outshine environmental ones, but, in fact, they were more prominent in the Different-Context condition. These results are in line with the prediction that context change affects the relative contribution of different cues to memory, with semantic cues playing a more prominent role in the Different-Context condition than in the Same-Context condition. Moreover, our results provide an important constraint to the theoretical proposal made by Unsworth et al. (2012) that changes in context do not affect the cuing of a retrieval attempt by the previously-recalled item. Like Unsworth et al. (2012), we found that cuing of an item by another is indeed not affected when indexed by temporal clustering. However, such cueing is affected when indexed by semantic clustering. These results are in line with predictions based on both eSAM and CMR, according to which, temporal and semantic clustering are driven by different underlying mechanisms. Hence, the findings that TCE is not sensitive to contextual change does not entail that semantic clustering will not be affected either.

The manipulation of context change may be simulated by both eSAM and CMR models. In eSAM, the poorer match between encoding and retrieval context cues in the Different-Context condition might be simulated by weakening the associations between items and the list context. In the CMR, the background music may be simulated as a source context that either matches or mismatches the encoding context in the Same-Context and Different-Context conditions, respectively. Future work, including simulation studies, is needed to determine which of the models provides a better account of the effects of context change on semantic clustering and other recall effects.

Can greater reliance on semantic clustering in the Different-Context condition be explained as a compensation strategy that participants use when contextual cues are meager? Previous work (Polyn et al., 2011) showed that semantic relations between words allow the memory system to bridge the temporal gap separating them (to compensate for the lack of temporal context cues). Similarly, it is possible that semantic relations between words can also compensate for the lack of environmental context. The ability of participants to compensate for a change of context by relying on a semantic retrieval strategy can also explain the lack of a significant Context Dependency Effect in the present experiment. In order to test this compensation hypothesis, we examined the correlation between correct recall and the Semantic Factor scores, for both conditions. We expected to see a negative correlation indicating that semantic organization is a form of compensation. However, in practice, a negative but insignificant correlation was obtained. A possible explanation for the lack of a significant correlation could be that there was not much between-participant variability in the Semantic Factor scores. Therefore, our data are not sufficient to support the compensation hypothesis.

An additional goal of the current study was to examine if the results reported in previous work (Unsworth et al., 2012) regarding the influence of context change on temporal organization, patterns of errors and serial position extends to a more ecologically valid condition, in which semantic organization can be relied upon (Gilboa and Marlatte, 2017). To this end, we examined the influence of context change on the Lag-CRP function, serial position curves, and pattern of errors. These measures did not differ between the Same-Context condition and the Different-Context condition. In addition, the Temporal Factor scores did not vary between conditions but were above chance in both conditions, indicating that participants used temporal clustering during recall. These results are consistent with previous work of the Context Dependency Effect (Unsworth et al., 2012). Our experiment extends these findings by showing that they also hold under more ecologically valid conditions, in which semantic organization can be relied upon.

A possible direction for future research would be to examine whether the patterns of greater reliance on the semantic organization in the Different-Context condition compared to the Same-Context condition will hold for change of context when the manipulation of context is internal. The results of previous research (Unsworth et al., 2012) suggests that external and internal context change represent fundamentally the same cognitive operations. We would thus expect to find more reliance on the semantic organization in the Different-Context condition compared to the Same-Context condition also when internal context is manipulated.

In conclusion, the present experiment expands our understanding of the Context Dependency Effect in episodic memory. Even though there was no statistically significant difference in the mean number of correctly recalled words as a function of context change, we showed that context change influenced the reliance on semantic clustering during recall. When participants are limited in their ability to rely on contextual reinstatement (as is the case in the Different-Context condition), they rely more on semantic organization in driving recall.

## Data availability statement

The original contributions presented in the study are included in the article/supplementary material, further inquiries can be directed to the corresponding author.

## Ethics statement

The studies involving human participants were reviewed and approved by Research Committee on Human Issues of the Department of Psychology, Ben-Gurion University of the Negev, Israel. Participants read and signed informed consent of willingness to participate in the study.

## Author contributions

DV wrote the first draft of the manuscript. AS organized the database. AS and ZR-G performed the statistical analysis. TS supervised and designed the research, and obtained funding. All authors wrote sections of the manuscript, contributed to the article, and approved the submitted version.

## Footnotes

^1^ http://memory.psych.upenn.edu/files/wordpools/PEERS_wordpool.zip

^2^ https://memory.psych.upenn.edu/Software
